# Prognostic Value of the Lung Immune Prognostic Index and an ECOG–Albumin–LIPI Nomogram in Metastatic NSCLC Patients Treated with Second- or Third-Line Nivolumab

**DOI:** 10.3390/jcm15176869

**Published:** 2026-09-04

**Authors:** Didem Divriklioğlu, İsmail Bayrakçı, Gizem Bakır Kahveci, İvo Gökmen, Dicle Yurdatap Koç, Ece Demirdelen, Ahmet Küçükarda, Muhammet Bekir Hacıoğlu, Bülent Erdoğan, Sernaz Topaloğlu

**Affiliations:** 1 Department of Medical Oncology, Edirne Sultan 1. Murat State Hospital, 22030 Edirne, Türkiye; 2 Department of Medical Oncology, Nevşehir State Hospital, 50300 Nevşehir, Türkiye; dr.ismaiilbayrakci@hotmail.com; 3 Department of Medical Oncology, Faculty of Medicine, Trakya University, 22030 Edirne, Türkiye; bakirkahvecigizem@gmail.com (G.B.K.); dicleyurdatap@gmail.com (D.Y.K.); demirdelen_ece@hotmail.com (E.D.); ahmetkucukarda22@gmail.com (A.K.); mbekirhacioglu@yahoo.com (M.B.H.); berdoga@hotmail.com (B.E.); sernaz.uzunoglu@gmail.com (S.T.); 4 Department of Medical Oncology, Faculty of Medicine, Çanakkale Onsekiz Mart University, 17100 Çanakkale, Türkiye; ivo_georgiev1@hotmail.com

**Keywords:** non-small cell lung cancer, nivolumab, Lung Immune Prognostic Index, albumin, ECOG

## Abstract

**Background/Objectives:** Clinical outcomes with immune checkpoint inhibitors (ICIs), such as nivolumab, vary among patients with metastatic non-small cell lung cancer (NSCLC). The Lung Immune Prognostic Index (LIPI) may help stratify prognosis. This study evaluated the prognostic significance of LIPI in patients receiving second- or third-line nivolumab and developed a nomogram for individualized survival estimation. **Methods:** This single-center retrospective study included 142 patients with metastatic NSCLC who received second- or third-line nivolumab between February 2022 and December 2024, after progression on platinum-based chemotherapy. LIPI was calculated from baseline values obtained within 14 days before nivolumab initiation, based on a derived neutrophil-to-lymphocyte ratio (dNLR) > 3 and lactate dehydrogenase (LDH) > 225 U/L (institutional upper limit of normal), classifying patients as good-, intermediate-, or poor-risk (0, 1, or 2 factors, respectively). Overall survival (OS) and progression-free survival (PFS) were estimated by the Kaplan–Meier method; independent prognostic factors were assessed by multivariable Cox regression, and a prognostic nomogram combining ECOG performance status, serum albumin, and LIPI was developed and internally validated. **Results:** By LIPI, 34.5%, 45.1%, and 20.4% of patients were at good, intermediate, and poor risk, respectively. Objective response and disease control rates were 29.6% and 55.6%. Median OS and PFS were 19.3/8.0/3.1 and 8.6/3.1/2.5 months across good, intermediate, and poor LIPI groups (log-rank *p* < 0.001). In multivariable analysis, ECOG 2 (hazard ratio [HR], 4.94), albumin per 1 g/dL (HR 0.27), and poor versus good LIPI (HR 3.74) were independently associated with OS. A nomogram combining these three factors showed acceptable discrimination (optimism-corrected Harrell C-statistic 0.742). **Conclusions:** LIPI was independently associated with prognosis in this cohort. Combining LIPI with ECOG and albumin may aid individualized risk assessment, pending external validation.

## 1. Introduction

The integration of immune checkpoint inhibitors (ICIs) into the therapeutic regimen for metastatic non-small cell lung cancer (NSCLC) has significantly changed its treatment paradigm. Nivolumab, an anti- Programmed Death-1 antibody, has shown a substantial improvement in overall survival (OS) compared to docetaxel in cases of advanced disease that progress following platinum-based chemotherapy. Consequently, it has become a standard treatment option for this patient group [1]. Clinical outcomes with ICIs are heterogeneous across patients, and identifying subgroups with a more or less favorable prognosis under later-line nivolumab remains clinically relevant, even though such prognostic associations alone cannot establish which patients derive differential treatment benefit from ICIs specifically.

PD-L1 (Programmed Death-Ligand 1) expression and tumor mutation burden are significant biomarkers employed in predicting treatment response; nonetheless, their independent application frequently proves inadequate [2]. This inadequacy may stem from intratumoral heterogeneity, challenges in acquiring adequate tissue samples, and variations in measurement methodologies.

In recent years, inflammation-based prognostic indices derived from routine hematological and biochemical parameters have been investigated for their prognostic associations with outcomes in advanced NSCLC, including in cohorts treated with immunotherapy. These indices include the pan-immune–inflammation value (PIV); the PIV–LDH-–ECOG-based (PILE) score, which integrates PIV, lactate dehydrogenase (LDH), and Eastern Cooperative Oncology Group (ECOG) performance status; the systemic immune–inflammation index (SII); and the platelet-to-lymphocyte ratio (PLR) [3,4,5]. Among these, the Lung Immune Prognostic Index (LIPI) combines the derived neutrophil-to-lymphocyte ratio (dNLR), a marker of systemic inflammation, with LDH, a marker associated with tumor burden and tissue injury. LIPI has been shown to stratify prognosis in patients with advanced NSCLC treated with ICIs, with poor-risk patients experiencing shorter OS and PFS [6]. However, inflammation-based indices alone may not fully capture host functional and nutritional reserve. Higher ECOG and lower serum albumin have also been associated with worse survival [7]. Integrating LIPI with ECOG and serum albumin in a nomogram may therefore provide a more individualized prognostic estimate.

Multiple studies have demonstrated the prognostic significance of LIPI in patients with metastatic NSCLC receiving ICIs [8]. LIPI classifies patients into broad good-, intermediate-, and poor-risk groups but may not capture all biological, functional, and nutritional differences within each group. Models that integrate complementary prognostic factors may therefore provide more individualized estimates than LIPI classification alone. The novelty of the present study lies in three elements: evaluation of LIPI within a relatively homogeneous, ICI-naive cohort restricted to second- or third-line nivolumab monotherapy, in contrast to the heterogeneous ICI-treated populations in which LIPI has previously been validated; assessment of LIPI specifically within this later-line, single-agent setting; and development of a preliminary ECOG–albumin–LIPI nomogram that translates the three-tier LIPI classification into individualized 6-, 12-, and 18-month survival probability estimates.

This study aimed to assess the prognostic significance of LIPI within a cohort of patients with metastatic NSCLC undergoing second- and third-line nivolumab monotherapy; because no non-ICI comparator was included, treatment-predictive utility was outside the scope of this analysis. Additionally, we constructed a nomogram to predict individual survival probabilities by integrating parameters identified as independent prognostic factors with the LIPI.

## 2. Materials and Methods

### 2.1. Study Design and Ethical Approval

This single-center, retrospective cohort study was conducted to evaluate the prognostic factors and survival of patients receiving nivolumab as a second- or third-line treatment for metastatic NSCLC. The study was reported in accordance with the STROBE (Strengthening the Reporting of Observational Studies in Epidemiology) statement for cohort studies, and the development and internal validation of the prognostic nomogram were reported in accordance with the TRIPOD (Transparent Reporting of a multivariable prediction model for Individual Prognosis Or Diagnosis) statement; the completed checklists are provided as Supplementary Material (Tables S1 and S2).

### 2.2. Study Population

Eligible patients were treated at the Department of Medical Oncology, Trakya University, and initiated nivolumab between February 2022 and December 2024. Inclusion criteria were histologically or cytologically confirmed metastatic NSCLC, progression after first-line platinum-based combination chemotherapy, and receipt of at least one dose of nivolumab as second- or third-line therapy. All patients had received platinum-based chemotherapy without an ICI as first-line therapy; the cohort was therefore ICI-naive at nivolumab initiation. Nivolumab was administered in the second line in 126 patients (88.7%) and in the third line in 16 (11.3%). The median interval from first-line initiation to nivolumab was 7.9 months. Systemic therapies administered after nivolumab were recorded descriptively and, being post-baseline events, were not entered as covariates (Table S3). In every patient, nivolumab initiation followed radiologically documented disease progression during or after first-line platinum-based chemotherapy; no toxicity-driven switch was identified. Exclusion criteria were a second primary malignancy, liver or kidney failure, diagnosed autoimmune disease, age < 18 years, or unavailable clinical or laboratory data. All patients underwent routine molecular testing for EGFR, ALK, and ROS1 alterations; no targetable alterations were detected, and therefore no patients were excluded on this basis. KRAS testing was not performed routinely in the entire cohort; however, among patients who were tested, no KRAS mutations were identified. Of 147 patients screened, 142 met the eligibility criteria and were included.

### 2.3. Data Collection

Clinical and pathological data were retrospectively obtained from electronic medical records and patient files. Age was calculated at nivolumab initiation. Baseline laboratory parameters were defined as the measurements closest to nivolumab initiation and obtained within the prespecified 14-day window. LDH and albumin were measured using an automated chemistry analyzer (Roche Hitachi Cobas 8000; Rotkreuz, Switzerland), and neutrophil and lymphocyte counts were measured using a hematology analyzer (Sysmex SE-9000; Kobe, Japan). ECOG was assessed before treatment initiation. Tumor PD-L1 expression was assessed immunohistochemically using the Ventana SP263 assay (Ventana Medical Systems; Tucson, AZ, USA) on the Ventana BenchMark XT platform (Ventana Medical Systems; Tucson, AZ, USA) and recorded as tumor proportion score (TPS).Treatment response and disease progression were assessed using computed tomography or cranial magnetic resonance imaging according to Response Evaluation Criteria in Solid Tumors (RECIST), version 1.1. Missing data were handled using multiple imputation as described in the Section 2.5.

### 2.4. Variables and Endpoints

This study evaluated age, gender, ECOG, smoking history, histological subtype, and the presence of liver, bone, brain, and adrenal metastases, alongside leukocyte, neutrophil, lymphocyte, monocyte, platelet, hemoglobin, albumin, LDH, and C-reactive protein levels at the initiation of nivolumab treatment as independent variables. Indices of systemic inflammation and prognosis were calculated using the baseline laboratory values. The neutrophil–lymphocyte ratio (NLR) was calculated by dividing the neutrophil count by the lymphocyte count, and the dNLR was calculated by dividing the neutrophil count by the difference between the leukocyte and neutrophil counts. The LIPI was established based on two components: a dNLR greater than 3 and LDH levels exceeding 225 U/L, which is the institutional upper limit of the normal range. Patients were categorized into three distinct risk groups: those without either component were classified as having a good-risk classification (factor 0), those with one component as having an intermediate-risk classification (factor 1), and those with both components as having a poor-risk classification (factor 2) [6].

In addition to LIPI, the PIV (calculated as neutrophils multiplied by monocytes and platelets, divided by lymphocytes), SII (calculated as platelets multiplied by neutrophils, divided by lymphocytes), and PLR (calculated as platelets divided by lymphocytes) were derived from the peripheral blood count at nivolumab treatment initiation. These indices were dichotomized at the median values of the cohort. The PILE score was categorized as low (0–1) or high (2–3) based on a composite scoring system that assigned one point each for PIV values above the median, LDH levels exceeding the upper limit of normal, and an ECOG of 2 or greater.

The primary endpoint was OS, defined as the interval from nivolumab initiation to death from any cause. Patients alive at the database-lock date were censored at their last documented follow-up. The secondary endpoint was progression-free survival (PFS), defined as the interval from nivolumab initiation to radiological progression or death from any cause, whichever occurred first. Patients without progression or death were censored at their last disease assessment. Treatment response was classified according to RECIST 1.1 as complete response, partial response, stable disease, progressive disease, or not evaluable. Objective response rate (ORR) was defined as the proportion of the entire cohort achieving complete or partial response, and disease control rate (DCR) as the proportion achieving complete response, partial response, or stable disease. Patients without a formal RECIST assessment were retained in the whole-cohort denominator; this conservative estimand should be distinguished from response-evaluable rates.

### 2.5. Statistical Analysis

Statistical analyses were performed using jamovi (version 2.6.44; The jamovi project, Sydney, Australia), JASP (version 0.19.3; JASP Team, University of Amsterdam, Amsterdam, The Netherlands), and Python (version 3.12; Python Software Foundation, Beaverton, OR, USA), with the lifelines package (version 0.30.3) for survival analyses and Matplotlib (version 3.10.8) for figure generation; multiple imputation (predictive mean matching), Rubin pooling, and bootstrap internal validation of the nomogram were performed in Python. The distribution of continuous variables was assessed using the Lilliefors-corrected Kolmogorov–Smirnov test, along with skewness and kurtosis. Normally distributed variables are summarized as mean ± standard deviation, and non-normally distributed variables are summarized as median (interquartile range [IQR]). Categorical variables are presented as counts and percentages.

The distribution of categorical variables across LIPI groups was compared using the Pearson chi-square or Fisher–Freeman–Halton test according to expected cell frequencies; effect size was expressed as Cramér’s V with 95% confidence intervals (CI) obtained from 2000 bootstrap replicates. Treatment response was classified according to RECIST 1.1. The 95% CIs for objective response and DCR were calculated using the Wilson method. Objective response was additionally analyzed with multivariable logistic regression including LIPI, age (per 10 years), and bone metastasis; Firth penalization was prespecified as a fallback in the event of separation. This analysis was performed in response to editorial review and is reported outside the two prespecified multiplicity families.

Survival was estimated using the Kaplan–Meier method, and groups were compared using the log-rank test. Median follow-up was estimated with the reverse Kaplan–Meier method, in which the censoring indicator is reversed so that patients alive at the database-lock date contribute their full observed follow-up time. Prognostic factors were examined using univariable and multivariable Cox proportional-hazards regression analyses. The covariates of the multivariable model—age, ECOG, histology, liver metastasis, PD-L1, albumin and LIPI—were prespecified in the analysis plan on clinical grounds before any model was fitted, and their number was constrained by the available number of events (10 degrees of freedom; events-per-variable ≈ 12.6). Candidate predictors were assembled before modeling from prognostic factors established in previously treated NSCLC receiving ICIs: age, sex, ECOG performance status, smoking status, histology, metastatic disease type, liver/bone/brain/adrenal metastases, PD-L1 status, treatment line, albumin, and LIPI. Age (per 10 years) and albumin (per 1 g/dL) were prespecified as continuous, ECOG and LIPI as categorical. The multivariable covariate set was fixed in the analysis plan on clinical grounds and applied identically to OS and PFS; univariable results (screening threshold *p* < 0.10) served only as supporting evidence, and no stepwise or other data-driven selection was used. The final model comprised 10 degrees of freedom against 126 deaths (events per variable ≈ 12.6). The nomogram specification (ECOG, albumin, LIPI) was likewise fixed before estimation as a parsimonious prediction model and was internally validated by bootstrap resampling with optimism correction. The same covariate set was used for the OS and PFS models so that the two analyses remain directly comparable. Missing PD-L1 data (25.4%) were handled by predictive mean matching multiple imputation (m = 30), including the Nelson-Aalen cumulative hazard estimate and the event indicator of the outcome; coefficients were pooled using Rubin’s rules. A complete case analysis was performed as a sensitivity analysis. The proportional-hazards assumption was tested using scaled Schoenfeld residuals, and multicollinearity was evaluated using the variance inflation factor. Multiple comparisons were corrected for using the Benjamini–Hochberg false discovery rate procedure within two prespecified test families: the coefficients of the confirmatory multivariable OS model and the exploratory univariable analyses of the PIV, PILE, SII, and PLR indices (four indices × two endpoints, k = 8).

A nomogram for OS was developed based on ECOG performance status (modeled categorically: 0, 1, 2), albumin (linear; linearity verified with restricted cubic splines), and LIPI. The linearity of the albumin association was assessed with restricted cubic splines (3 and 4 knots) against the linear specification, both in the nomogram model and in the full multivariable model under multiple imputations. Discrimination was assessed using the Harrell C-statistic and calibration with the calibration slope and 12-month Brier score; internal validation was performed using 1000 bootstrap replicates with optimism correction. A two-sided *p*-value ≤ 0.05 was considered statistically significant.

### 2.6. Use of Artificial Intelligence Tools

Artificial intelligence-based language editing tools (OpenAI’s ChatGPT, version GPT-5, and Paperpal (accessed August 2026) were used for language editing and readability improvement. These tools were not used for data collection, statistical analysis, or interpretation of results. All AI-assisted revisions were carefully reviewed and verified by the authors, who took full responsibility for the accuracy, originality, and scientific integrity of the manuscript.

## 3. Results

### 3.1. Patient Characteristics

**Figure 1:**
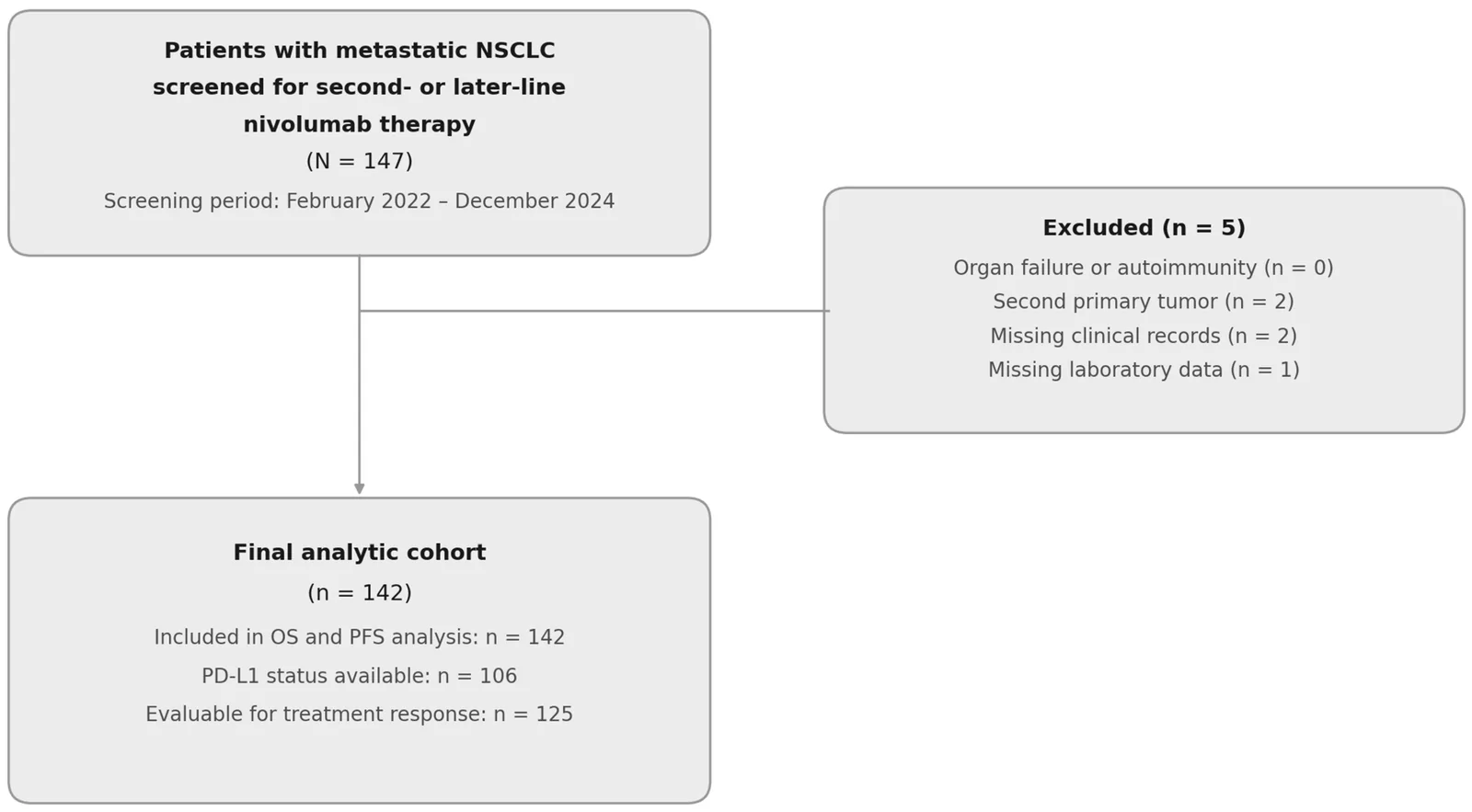
Patient selection flowchart. NSCLC, non-small cell lung cancer; OS, overall survival; PFS, progression-free survival; PD-L1, programmed death-ligand 1.

A total of 147 patients with metastatic NSCLC who received second- or third-line nivolumab were screened. Five patients were excluded: none for organ failure or autoimmune disease, two for a concurrent second primary tumor, two for missing clinical records, and one for missing laboratory data, leaving 142 patients in the final cohort (Figure 1). OS and PFS were analyzed in the entire cohort. PD-L1 results were available for 106 patients, and formal RECIST response assessment was available for 125 patients. The patient-selection process is shown in Figure 1.

The median age was 65.4 years (IQR 60.1–69.7), and 52.1% were aged ≥65 years; 86.6% were male, and 64.1% had an ECOG of 0. Within the patient cohort, squamous cell carcinoma was present in 50.7% of the cases, whereas adenocarcinoma was detected in 43.7% of the cases. Additionally, 66.9% of the patients exhibited de novo metastatic disease, and 88.7% received nivolumab as a second-line therapy (Table 1).

The diagnosis was established histologically in 126 patients (88.7%) and cytologically in 16 (11.3%) (Table 1). Reasons for nivolumab discontinuation are summarized in Table S4, the most frequent being disease progression (*n* = 80, 56.3%).

Overall, the cohort was predominantly male, elderly, and squamous-histology-predominant, with the majority receiving nivolumab as second-line therapy following de novo metastatic disease.

### 3.2. LIPI Risk Stratification

According to the LIPI, 49 patients (34.5%) were classified as good, 64 (45.1%) as intermediate, and 29 (20.4%) as poor risk. Most baseline characteristics were comparable across the LIPI groups; only the proportion of patients aged ≥65 years (*p* = 0.047) and the frequency of bone metastasis differed between groups (26.5%, 39.1%, and 65.5%, respectively; *p* = 0.003, Cramér’s V = 0.286). No statistically significant differences were observed in sex, ECOG, histology, metastatic disease type, or PD-L1 status (Table 2).

In summary, LIPI risk groups were well balanced with respect to most baseline characteristics, apart from age and bone metastasis, supporting comparability across risk strata.

### 3.3. Treatment Response and Survival Outcomes

Using the prespecified whole-cohort denominator (*n* = 142), the ORR was 29.6% (42 patients; 95% CI, 22.7–37.5) and the DCR was 55.6% (79 patients; 95% CI, 47.4–63.5); no complete responses were observed. Across the good-, intermediate-, and poor-risk LIPI groups, response distribution differed significantly (*p* = 0.003), while ORR decreased from 42.9% to 26.6% and 13.8% (*p* = 0.019), and DCR decreased from 75.5% to 46.9% and 41.4% (*p* = 0.002), respectively (Table 3). In the response-evaluable population (*n* = 125), ORR was 33.6% (95% CI 25.9–42.3), and DCR was 63.2% (95% CI 54.5–71.1). Reasons for non-evaluability and the baseline comparison with evaluable patients are given in Table S5. In multivariable logistic regression adjusting for age and bone metastasis, LIPI remained independently associated with objective response (intermediate vs. good OR 0.32, 95% CI 0.13–0.76, *p* = 0.012; poor vs. good OR 0.14, 95% CI 0.03–0.49, *p* = 0.004; likelihood-ratio test *p* = 0.003).

**Figure 2:**
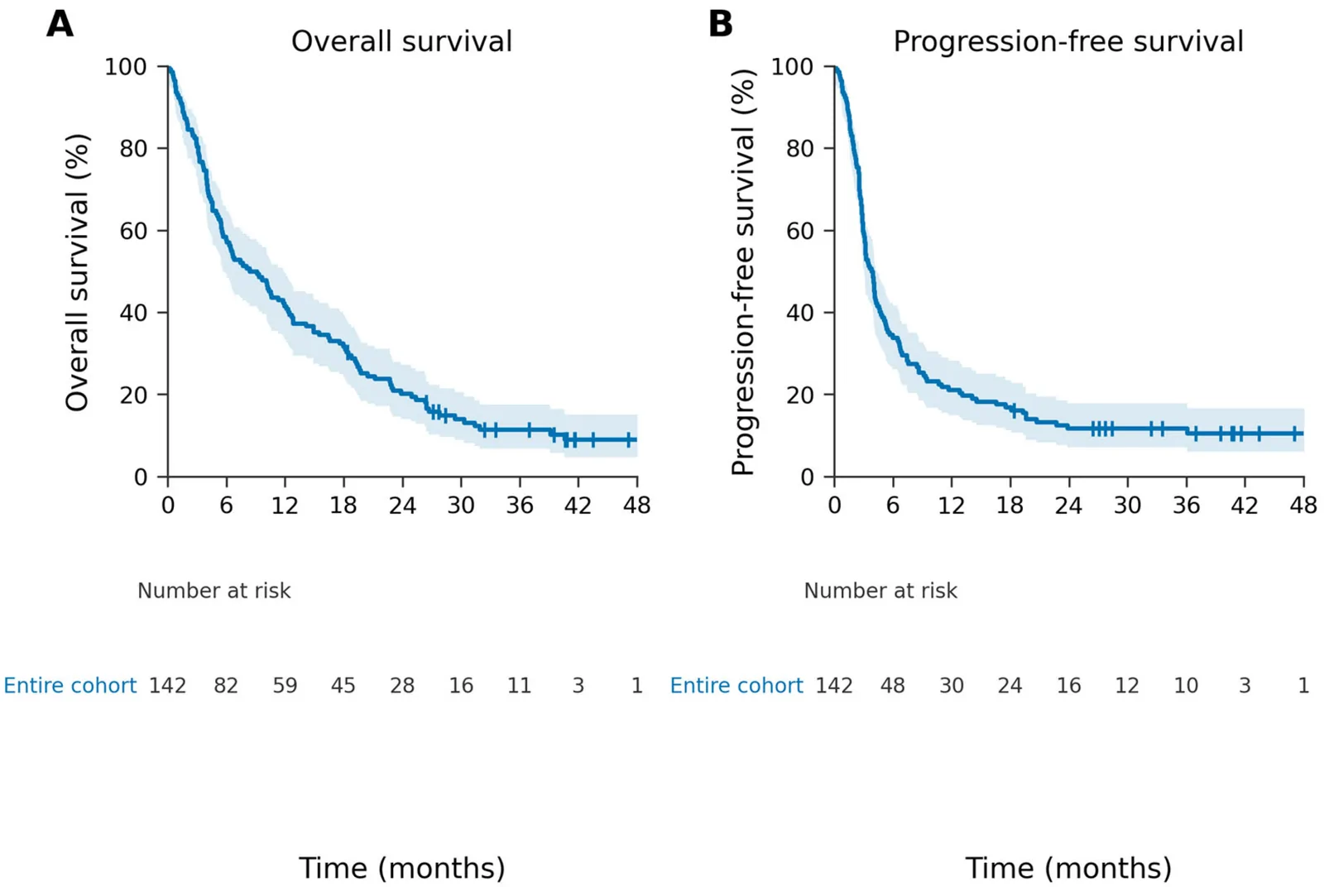
Kaplan–Meier estimates of overall survival (**A**) and progression-free survival (**B**) in the entire cohort. Curves are shown with their 95% confidence intervals (shaded bands); tick marks denote censored observations, and the number of patients at risk is given below each panel. Median OS was 8.3 months (95% CI 5.7–11.9) and median PFS was 3.8 months (95% CI 3.0–4.4), over a median follow-up of 40.6 months (*n* = 142). CI, confidence interval.

**Figure 3:**
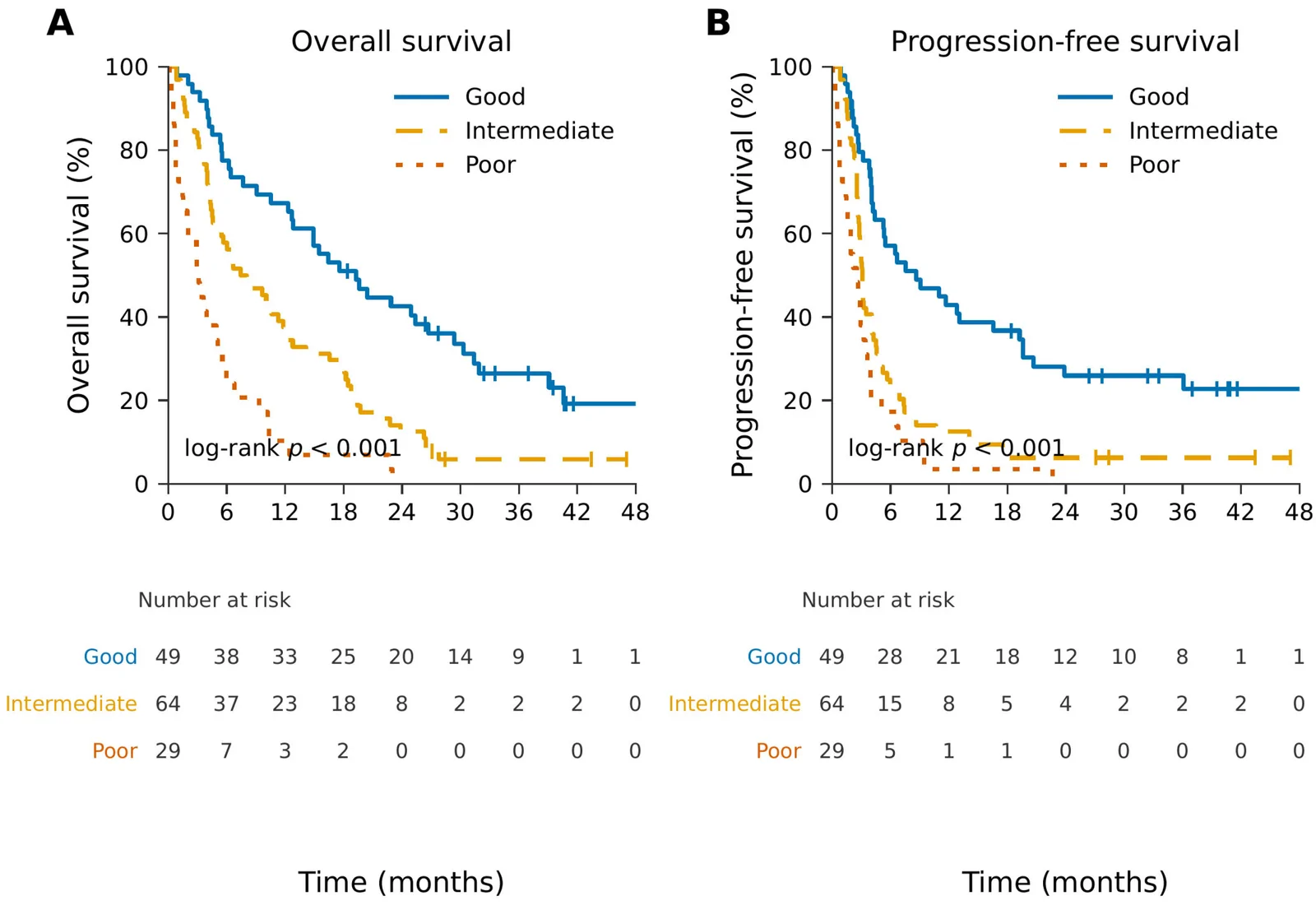
Kaplan–Meier estimates of overall survival (**A**) and progression-free survival (**B**) stratified by the Lung Immune Prognostic Index (LIPI). Patients were classified as good (*n* = 49), intermediate (*n* = 64), or poor (*n* = 29). Between-group differences were assessed by the log-rank test (*p* < 0.001 for both endpoints). Median OS was 19.3, 8.0, and 3.1 months and median PFS was 8.6, 3.1, and 2.5 months in the good-, intermediate-, and poor-risk groups, respectively. Tick marks denote censored observations; the number of patients at risk is shown below each panel.

At a median follow-up of 40.6 months, 126 of the 142 patients (88.7%) had died, and 16 (11.3%) were alive at the database-lock date. Follow-up continued after nivolumab discontinuation until death or the database-lock date, which is why the median follow-up substantially exceeds the duration of nivolumab exposure. The reverse Kaplan–Meier estimate of median follow-up was 40.6 months (95% CI 32.4–41.6) in the good and 43.5 months (95% CI 27.1–47.1) in the intermediate-risk LIPI group; in the poor-risk group, the median follow-up was not estimable by this method because all 29 patients died during follow-up (observed follow-up range 0.1–23.0 months). The median OS was 8.3 months (95% CI 5.7–11.9), and the median PFS was 3.8 months (95% CI 3.0–4.4) (Figure 2). In the LIPI group, the median OS was 19.3, 8.0, and 3.1 months, and the median PFS was 8.6, 3.1, and 2.5 months in the good-, intermediate-, and poor-risk groups, respectively, with a statistically significant difference for both endpoints (log-rank *p* < 0.001) (Figure 3). The 12-month OS rates were 67%, 36%, and 10% in the good-, intermediate-, and poor-risk groups, respectively (Supplementary Table S6).

Collectively, these findings demonstrate a clear stepwise decline in treatment response, PFS, and OS across increasing LIPI risk categories.

### 3.4. Prognostic Factors and Nomogram Performance

**Figure 4:**
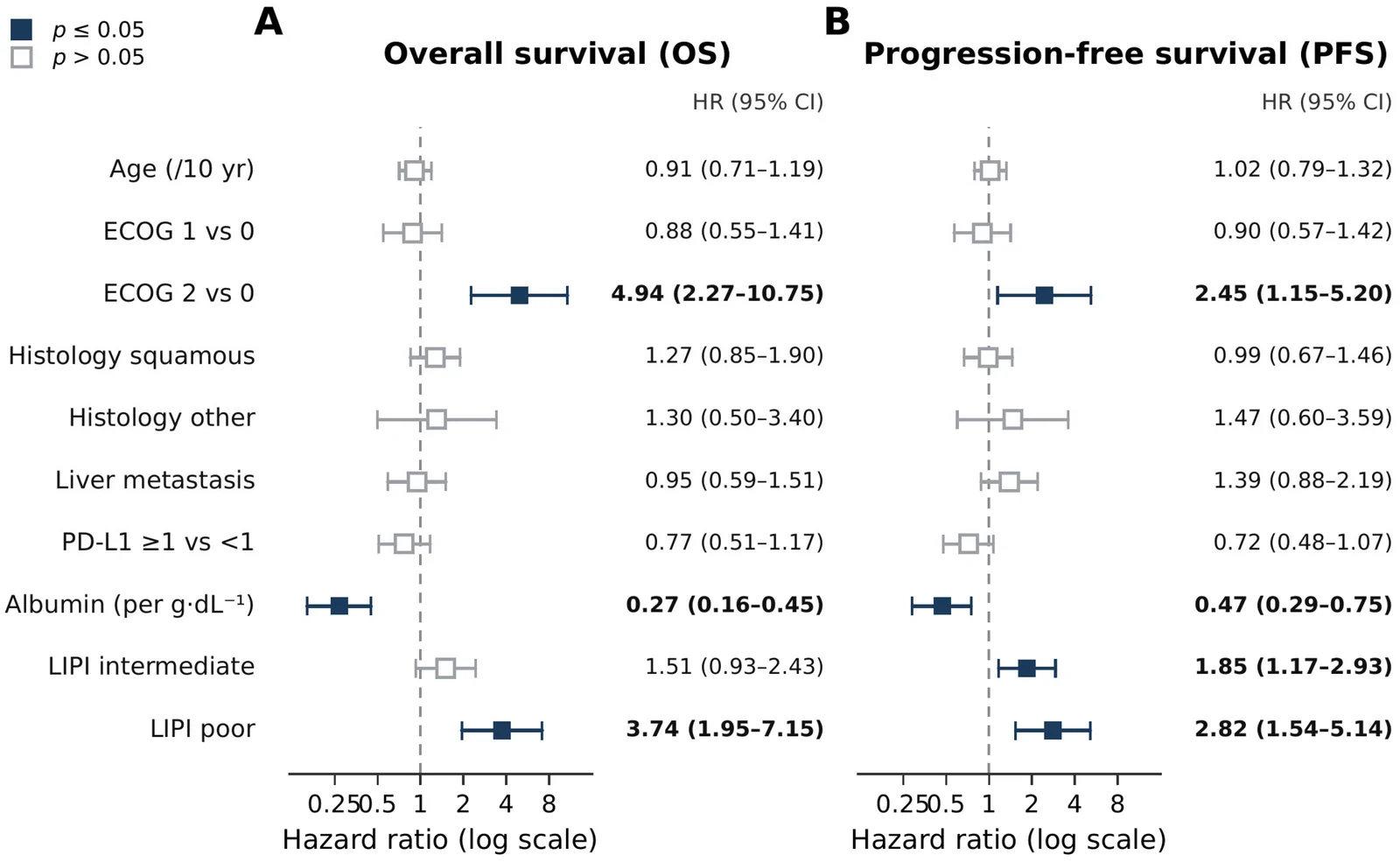
Forest plots of the multivariable Cox proportional-hazards models for overall survival (**A**) and progression-free survival (**B**). Squares represent adjusted hazard ratios and horizontal lines represent the corresponding 95% confidence intervals; the vertical dashed line marks a hazard ratio of 1. Filled squares indicate *p* ≤ 0.05 and open squares indicate *p* > 0.05. Bold HR values indicate statistically significant associations (*p* ≤ 0.05), consistent with filled squares. Both models were adjusted for age, ECOG performance status, histology, liver metastasis, PD-L1 status, serum albumin, and LIPI (10 degrees of freedom). Missing PD-L1 values were handled by multiple imputation (predictive mean matching, m = 30) with estimates pooled by Rubin’s rules; a complete-case analysis (*n* = 106) was consistent. The proportional-hazards assumption was confirmed using scaled Schoenfeld residuals, and multicollinearity was excluded (all VIF < 2). Hazard ratios are plotted on a logarithmic scale. CI, confidence interval; ECOG, Eastern Cooperative Oncology Group; HR, hazard ratio; LIPI, Lung Immune Prognostic Index; PD-L1, programmed death-ligand 1.

In univariable Cox analyses, ECOG 2, lower albumin, poor LIPI risk, and PD-L1 expression ≥ 1% were associated with OS. Among the exploratory indices, higher PIV, PILE, and SII were associated with OS, whereas PLR was not (*p* = 0.055); higher PIV and PILE were also nominally associated with PFS (Figure S1). After Benjamini–Hochberg correction within this prespecified eight-test family, the associations of PIV, PILE, and SII with OS and of PILE with PFS remained significant (q ≤ 0.007), whereas the nominal associations of PIV with PFS and of PLR with OS did not (q = 0.073 for both). In the multivariable OS model, ECOG 2 versus 0 (HR, 4.94; 95% CI, 2.27–10.75; *p* < 0.001), albumin per 1 g/dL increase (HR, 0.27; 95% CI, 0.16–0.45; *p* < 0.001), and poor versus good LIPI risk (HR, 3.74; 95% CI, 1.95–7.15; *p* < 0.001) remained independently associated with outcome; the intermediate LIPI group did not differ significantly from the good-risk group (*p* = 0.094). In the multivariable PFS model, ECOG 2 (HR, 2.45; *p* = 0.020), albumin (HR, 0.47; *p* = 0.002), intermediate LIPI (HR, 1.85; *p* = 0.008), and poor LIPI (HR, 2.82; *p* < 0.001) were independently associated with outcome (Figure 4; Table S7). Albumin values ranged from 2.6 to 4.9 g/dL (28 patients [19.7%] below 3.5 g/dL). Restricted cubic splines provided no evidence of nonlinearity (3-knot spline vs. linear term, *p* = 0.098 in the nomogram model and pooled *p* = 0.061 in the full multivariable model); in a categorical sensitivity analysis, albumin < 3.5 g/dL carried an adjusted HR of 2.63 (95% CI 1.65–4.20). Complete systemic treatment history is presented in Table S3. In a sensitivity analysis, after additionally adjusting for treatment line and the interval from first-line initiation to nivolumab, neither variable was associated with OS (*p* = 0.777 and *p* = 0.507), and the principal associations were unchanged.

**Figure 5:**
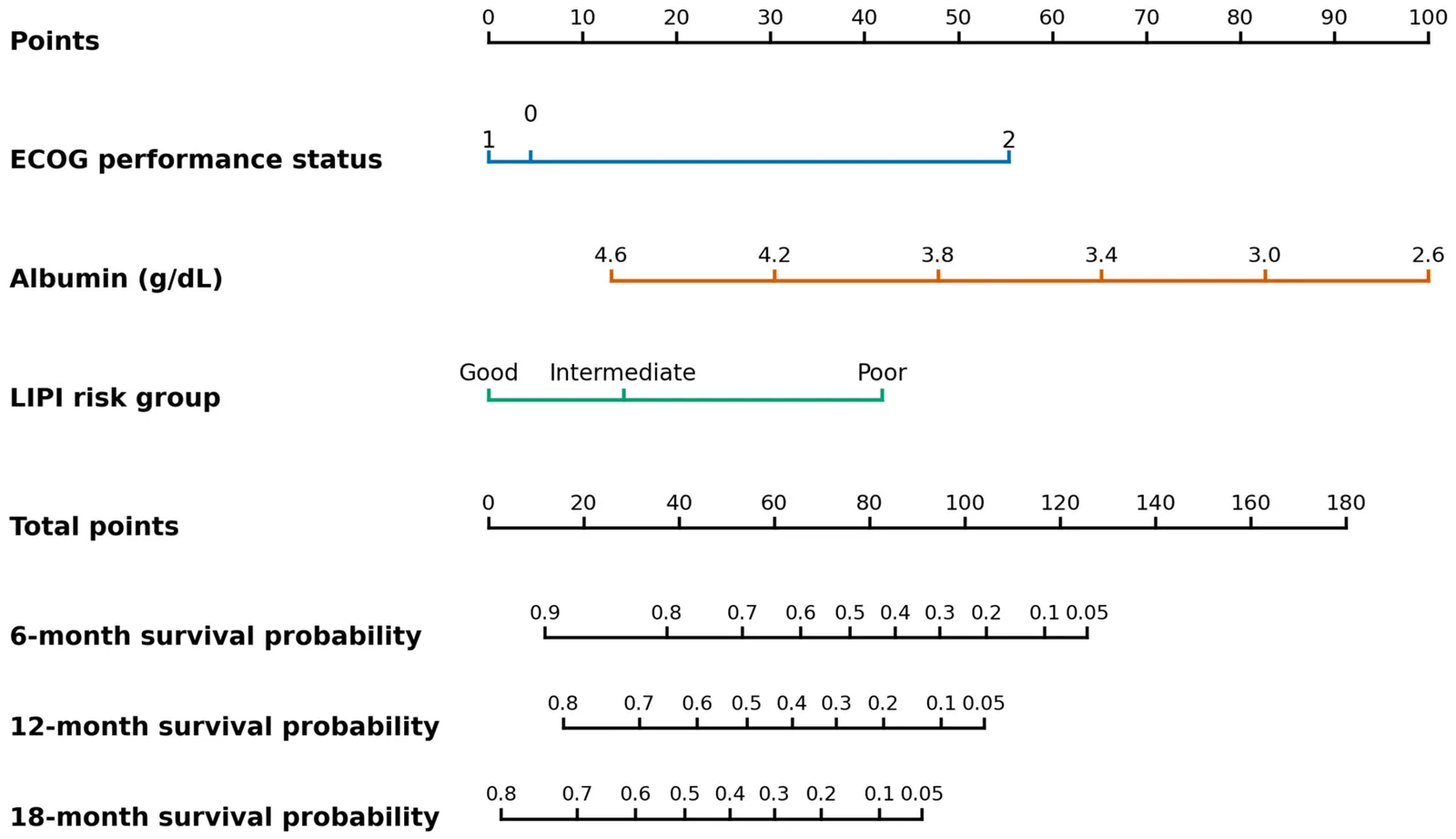
Nomogram for predicting 6-, 12-, and 18-month overall survival. The nomogram is based on a Cox model incorporating ECOG performance status (entered as a categorical variable: 0, 1, 2), serum albumin, and LIPI. For an individual patient, a vertical line is drawn from each predictor to the “Points” axis to read off the corresponding points; the sum is located on the “Total points” axis, and a downward vertical line yields the predicted survival probability at each time point. The model showed an apparent Harrell C-index of 0.752 (optimism-corrected 0.742). ECOG, Eastern Cooperative Oncology Group; LIPI, Lung Immune Prognostic Index.

For the OS nomogram incorporating ECOG, albumin, and LIPI, the optimism-corrected Harrell C-statistic was 0.742 (95% CI, 0.703–0.781), indicating acceptable internal discrimination. The optimism-corrected calibration slope was 0.909 (95% CI, 0.681–1.136), and the 12-month Brier score was 0.153. Across tertiles of the linear predictor, observed 12-month OS ranged from 10% to 82% (Figure 5 and Figure S2; Table 4). This model should be regarded as a preliminary prognostic tool; its performance requires confirmation in external cohorts before any clinical application.

In the sensitivity analysis modeling patients with unknown PD-L1 expression as a separate category, no statistically significant difference was observed between the unknown and PD-L1 < 1 groups for OS (*p* = 0.239) or PFS (*p* = 0.095); PD-L1 ≥ 1 remained associated with longer OS (HR: 0.62, *p* = 0.025) and PFS (HR: 0.58, *p* = 0.010) (Supplementary Table S8). The multiplicative LIPI × PD-L1 interaction was not significant (pooled Wald test, *p* = 0.620). In joint stratification among patients with known PD-L1 (*n* = 106), LIPI retained a significant survival gradient both in PD-L1-negative and in PD-L1-positive patients (log-rank *p* = 0.002 and *p* = 0.001, respectively), although the stratum-specific estimates are based on small numbers and carry wide confidence intervals (Table S9). In this subset, the nomogram linear predictor discriminated substantially better than PD-L1 alone (C-statistic 0.740 vs. 0.546), and adding PD-L1 to the nomogram did not improve model fit (*p* = 0.141).

Taken together, ECOG, albumin, and LIPI emerged as independent prognostic factors, and their combination in a nomogram provided acceptable internally validated discrimination for individualized survival prediction.

## 4. Discussion

This study demonstrated that pre-treatment LIPI risk stratification significantly distinguished both OS and PFS among patients receiving nivolumab as a second- or third-line therapy for metastatic NSCLC. Within the multivariate model, the poor LIPI risk group exhibited an approximately fourfold increase in the risk of mortality compared to the good-risk group, with a hazard ratio (HR) of 3.74. In addition to LIPI, ECOG and serum albumin were also identified as independent predictors of survival; the nomogram based on these three variables showed acceptable discrimination in predicting individual risk (C-statistic, 0.742). LIPI captures systemic inflammation and a biochemical correlate of tumor burden, whereas ECOG reflects functional status and albumin reflects nutritional and systemic reserve; these represent conceptually distinct physiological domains, and inflammation-based indices alone may not fully capture host functional and nutritional condition. The combined model is therefore intended to provide individualized numeric survival estimates rather than a categorical risk tier alone. The incremental value of the nomogram lies in this individualization, not in a claim of superior discrimination over LIPI, and external validation remains necessary before clinical application. The findings suggest that the LIPI, which is derived from commonly accessible laboratory parameters, in conjunction with a nomogram evaluating patients’ functional and nutritional status, can aid in the prognostic classification of this patient cohort. Mezquita et al. first defined LIPI and demonstrated that, among patients with advanced NSCLC receiving ICIs, a high LIPI score correlated with reduced OS and PFS. This association was corroborated by subsequent large-scale meta-analyses [6,8]. In our study, the survival difference observed among the LIPI risk groups was significant: the median OS was 19.3, 8.0, and 3.1 months in the good-, intermediate-, and poor-risk groups, respectively, and the PFS was 8.6, 3.1, and 2.5 months (log-rank *p* < 0.001), consistent with the results of previous studies. In a study conducted by Moratiel Pellitero et al., which assessed the factors influencing the efficacy of immunotherapy in metastatic NSCLC, the reported OS was 27 months for the LIPI good-risk group, 8 months for the intermediate-risk group, and 3 months for the poor-risk group [9]. Carril-Ajuria et al. demonstrated a significant survival advantage, with an HR of 0.47, in patients with metastatic renal cell carcinoma in the good LIPI group who received nivolumab. This finding supports the prognostic value of the LIPI score, independent of tumor type [10]. Gradual differences in survival rates among LIPI risk groups have been documented in studies primarily involving elderly patients and cohorts with small-cell lung cancer [11,12]. The consistent survival gradient observed across LIPI risk groups in our cohort and in previous studies supports the reproducibility of the prognostic association between LIPI and survival in this clinical setting. However, given the single-center retrospective design of the present study, external validation in independent cohorts remains necessary before LIPI-based risk stratification can be considered for individual clinical decision-making.

The LIPI classification was also associated with treatment response; the ORR declined from 42.9% to 13.8% in the good-risk group, while the DCR decreased from 75.5% to 41.4% (*p* = 0.019 and *p* = 0.002, respectively). The link between systemic inflammation and immunotherapy response is supported by studies showing that inflammatory biomarkers, such as dNLR and albumin, are associated with response and survival in patients with metastatic NSCLC receiving anti-PD-1 therapy [13].

The mechanistic interpretations discussed here are based on existing literature rather than on direct measurements within the present cohort. Given the retrospective design, we did not directly evaluate tumor hypoxia, glycolytic activity, tumor-associated neutrophils, lymphocyte function, immune-cell infiltration, or cytokine profiles. Therefore, these pathways should be considered plausible biological explanations for the observed associations with LIPI rather than mechanisms directly demonstrated by our study. Inflammation has been reported to contribute to cancer development, primarily through genotoxic damage, irregular cellular proliferation, and facilitation of invasion and metastasis. Experimental studies have shown that neutrophils can secrete chemokines and cytokines that may promote an immunosuppressive tumor microenvironment. Tumor cells have similarly been reported to secrete granulocyte-colony-stimulating factors, which may further enhance neutrophil accumulation around the tumor; this bidirectional interaction has been proposed to facilitate tumor proliferation and progression [14]. Lymphocytes are considered essential for the recognition and destruction of tumor cells and may thereby facilitate the antitumor effects of immunotherapy. A high dNLR, a component of LIPI, is considered a surrogate marker of a neutrophil-predominant inflammatory state [15]. LDH, the other component of LIPI, is widely used as a marker of tumor burden and cellular injury in patients with cancer. Elevated LDH levels have also been proposed to reflect increased glycolytic activity and tumor necrosis associated with hypoxic conditions [16].

In tumors exhibiting elevated glycolytic activity, the antitumor immune response may be inhibited as a result of glucose consumption, lactate accumulation, and acidosis within the tumor microenvironment [17]. Hypoxia-inducible factor 1-alpha activation has been reported to increase VEGF expression in tumor tissue, potentially contributing to abnormal neovascular structures and thereby limiting infiltration of antitumor immune cells into the tumor tissue [18]. The neutrophil-predominant inflammatory state, along with an increased tumor burden as indicated by elevated dNLR and LDH levels, is proposed to suppress the antitumor immune response, which may contribute to a generally worse prognosis irrespective of the treatment received [12,13]. This mechanism may explain the decreased response rates and lower survival observed in individuals categorized within the poor LIPI group.

Serum albumin was independently associated with OS; each 1 g/dL increase was associated with a lower hazard of death (HR, 0.27). This finding is consistent with evidence that pretreatment albumin and early albumin changes are associated with outcomes among patients with advanced lung cancer receiving ICI monotherapy, although this association may be attenuated with chemoimmunotherapy [19]. Evidence from advanced thymic carcinoma similarly suggests that albumin and LIPI provide complementary prognostic information [20]. Hypoalbuminemia in cancer may reflect systemic inflammation, malnutrition, and cachexia [21,22]. Preclinical findings also suggest potential links between hypoalbuminemia, macrophage metabolism, and resistance to immune checkpoint blockade [23]. Albumin is also incorporated into inflammation- and nutrition-based indices such as PNI, ALI, and GPS [24,25,26]. However, as each index reflects a different pathophysiological mechanism, it remains unclear which is superior in the context of immunotherapy. In our cohort, the fact that albumin maintained its independence in the multivariate model together with LIPI and that the nomogram based on the three variables exhibited acceptable discriminatory power (optimism-adjusted C-statistic 0.742; calibration slope 0.909) suggests that albumin may gain clinical value when used in conjunction with complementary indices rather than as an isolated parameter. Poor performance status (ECOG 2) was an independent predictor associated with an approximately five-fold (HR 4.94) increased risk of OS and a two-and-a-half-fold (HR 2.45) increased risk of PFS, respectively. The ECOG is a strong prognostic factor in patients with NSCLC treated with immunotherapy [27]. In a systematic review and meta-analysis by Tomasik et al., covering 67 studies and 26,442 patients, survival outcomes were shown to be significantly worse in patients with PS ≥ 2 than in those with PS ≤ 1; pooled HR were reported as 2.76 and 2.17 for OS and PFS, respectively [28]. Similarly, in a cohort of 429 patients of Chinese origin, Zhao et al. showed that ECOG 2–3 was an independent risk factor for OS, with an HR of 4.909 (95% CI 2.838–8.493; *p* < 0.001) [14]. In our study, the HR (2.45) obtained from the PFS analysis showed a close parallel with the pooled estimate of meta-analytic studies (2.17); while the HR value (4.94) we determined for OS surpassed the combined estimate of existing meta-analyses (HR: 2.76) and is in significant agreement with the single-center cohort data reported by Zhao et al. (HR: 4.909). This discrepancy can be explained by the limited number of patients with ECOG 2, the single-center retrospective design, and the fact that impairment in performance status can stem from various causes, such as comorbidities, age-related functional limitations, high tumor burden, or rapidly progressive disease.

In the univariate analysis, the exploratory variables PIV, PILE, and SII levels were associated with OS, with HRs of 1.84, 2.81, and 1.70, respectively. PLR was not significantly associated with OS (HR 1.41, 95% CI 0.99–2.01, *p* = 0.055). The PILE score was also associated with PFS after correction for multiplicity, whereas the association of PIV with PFS was nominal only. The prognostic importance of these indices aligns with a meta-analysis that demonstrated a correlation between C-reactive protein-based inflammatory markers and survival outcomes in lung cancer immunotherapy [29]. PIV, PILE, SII, and PLR were not entered into the multivariable models because they are structurally, and not merely statistically, redundant with covariates already in the model. PIV, SII, and PLR are all derived from the same neutrophil, lymphocyte, and platelet counts that generate the dNLR component of LIPI, while PILE is a composite score in which two of the three components—LDH above the upper limit of normal and ECOG ≥ 2—are already represented in the model, the former through LIPI and the latter as an independent covariate. Entering the four scores would also have increased the model from 10 to 14 degrees of freedom and reduced the events-per-variable ratio from 12.6 to 9.0, below the conventional threshold of 10. These indices were therefore prespecified as exploratory and interpreted only at the univariable level with Benjamini–Hochberg correction, and their independent contributions cannot be determined from the present data.

Some patient- and tumor-related features that have been shown to have prognostic significance in the literature did not reach independent significance in our study’s multivariate model. While PD-L1 expression (≥1 vs. <1) was associated with both OS and PFS in the univariate analysis (OS HR 0.64), no independent association was detected in the multivariate analysis (HR 0.77, *p* = 0.227). This result is consistent with the findings of a review highlighting that PD-L1 alone is insufficient for predicting the benefit of immunotherapy and should be evaluated in conjunction with other biomarkers [30]. Similarly, liver metastasis (HR 1.67), which was associated with PFS in the univariate analysis, lost its independence in the multivariate model (*p* = 0.157), suggesting that its effect may be attributed to the variance shared with LIPI and other covariates. The difference between our findings and those of a study that reported liver metastasis as an independent prognostic factor [14] can be explained by the sample size, covariate selection, and collinearity between variables.

In the multivariate model, the LIPI intermediate-risk group did not reach statistical significance for OS (*p* = 0.094), while both the intermediate and poor-risk groups were independent predictors of PFS; this difference may reflect the intermediate prognostic position of the middle group and its statistically significant difference depending on the outcome.

LIPI can be calculated from routine blood counts and LDH measurements without additional invasive procedures or laboratory costs. In patients receiving second- or later-line nivolumab, pretreatment LIPI may support prognostic counseling and risk stratification. The proposed nomogram combines LIPI with ECOG and albumin to provide individualized survival estimates beyond broad risk groups. Its apparent prognostic separation is encouraging, but external validation and assessment of clinical utility are required before it can be used to guide patient management.

Strengths of this study include its focus on a relatively homogeneous population receiving second- or third-line nivolumab monotherapy, adjustment for clinically relevant covariates, multiple imputation of missing PD-L1 data with complete-case sensitivity analysis, and bootstrap internal validation of the nomogram. Several limitations should be acknowledged. This study included only patients treated with second- or third-line nivolumab, without a comparator cohort receiving a non-ICI therapy (e.g., docetaxel). Consequently, the associations reported here establish the prognostic value of LIPI within this ICI-treated population but do not establish predictive value. Formal evaluation of predictive utility would require a treatment-by-LIPI interaction test in a design that includes a non-ICI comparator arm. The study period (February 2022–December 2024) preceded the expansion of reimbursement for first-line chemo-immunotherapy in Turkey in July 2025. During the study period, first-line chemo-immunotherapy was not reimbursed, and nivolumab reimbursement was limited to patients without prior exposure to ICIs. Therefore, the generalizability of our findings may be limited in contemporary healthcare settings where first-line chemo-immunotherapy is routinely used. KRAS testing was not performed uniformly across the entire cohort; therefore, the possibility that undetected KRAS alterations may have contributed to heterogeneity within the patient population cannot be excluded. Exclusion criteria for organ failure and autoimmune disease were prespecified because LDH, albumin, and systemic inflammatory markers, including those incorporated into the LIPI, may be influenced by non-malignancy-related conditions independently of tumor biology. However, no patients met these exclusion criteria in the present cohort (Figure 1), suggesting that their application did not materially restrict the study population or introduce selection bias. The single-center retrospective design introduces risks of selection bias, residual confounding, and incomplete documentation. The cohort size was moderate, and the ECOG 2 (*n* = 9) and third-line treatment (*n* = 16) subgroups were small, limiting estimate precision. Seventeen patients lacked formal RECIST 1.1 response assessment; because ORR and DCR used the entire cohort as the denominator, these estimates are conservative and differ from response-evaluable rates. The predominantly male population and high proportion of squamous histology may limit generalizability. Finally, the nomogram underwent internal validation only. External validation in independent, preferably multicenter prospective cohorts is required before clinical implementation.

## 5. Conclusions

Pretreatment LIPI was independently associated with OS, PFS, and treatment response among patients receiving second- or third-line nivolumab for metastatic NSCLC, with a clear stepwise survival gradient across the good-, intermediate-, and poor-risk groups. These findings establish the prognostic, not treatment-predictive, value of LIPI within this nivolumab-treated cohort; a comparator cohort and formal treatment-by-LIPI interaction analysis would be required to demonstrate predictive utility. ECOG and serum albumin provided additional, independent prognostic information beyond LIPI alone. The resulting ECOG–albumin–LIPI nomogram, based entirely on routinely available clinical and laboratory parameters, demonstrated acceptable internally validated discrimination (Harrell C-statistic 0.742) and may help refine individualized prognostic counseling. Given the single-center, retrospective design of this study and the absence of external validation, these findings should be regarded as hypothesis-generating; prospective, preferably multicenter, validation is required before the nomogram can be recommended for routine clinical decision-making.

## Figures and Tables

**Table 1:** Baseline demographic, clinical, and laboratory characteristics of the study cohort.

Variables	All Patients (*n* = 142)
**Demographic characteristics**	
**Age, years**	65.4 (60.1–69.7)
**Age ≥ 65, present**	74 (52.1)
**Sex**	
Female	19 (13.4)
Male	123 (86.6)
**ECOG performance status**	
0	91 (64.1)
1	42 (29.6)
2	9 (6.3)
**Smoking status**	
Current smoker	80 (56.3)
Former smoker	50 (35.2)
Never smoker	12 (8.5)
**Disease characteristics**	
**Histology**	
Adenocarcinoma	62 (43.7)
Squamous cell carcinoma	72 (50.7)
Other	8 (5.6)
**Method of diagnosis**	
Histological	126 (88.7)
Cytological	16 (11.3)
**Metastatic disease type**	
De novo metastatic	95 (66.9)
Recurrent metastatic	47 (33.1)
**Liver metastasis, present**	29 (20.4)
**Bone metastasis, present**	57 (40.1)
**Brain metastasis, present**	24 (16.9)
**Adrenal metastasis, present**	37 (26.1)
**PD-L1 TPS**	
<1	55 (38.7)
1–5	8 (5.6)
5–10	5 (3.5)
10–50	21 (14.8)
>50	17 (12.0)
Unknown	36 (25.4)
**Treatment**	
**Nivolumab line**	
Second	126 (88.7)
Third	16 (11.3)
**Time from diagnosis to nivolumab, months**	9.4 (6.0–16.1)
**Laboratory at nivolumab initiation**	
**Leukocytes, µL**	8449 ± 3424
**Neutrophils, µL**	5340 (3618–7550)
**Lymphocytes, µL**	1375 (870–1810)
**Monocytes, µL**	720 (575–910)
**Platelets, µL**	262,000 (194,750–352,750)
**Hemoglobin, g/dL**	11.1 ± 1.7
**Albumin, g/dL**	3.90 (3.60–4.30)
**Lactate dehydrogenase, U/L**	242.5 (193.0–352.8)
**C-reactive protein, mg/L (*n* = 134)**	23.5 (6.0–47.7)
**NLR**	4.10 (2.74–6.25)
**dNLR**	2.24 (1.67–3.30)
**Prognostic scores**	
**LIPI**	
Good	49 (34.5)
Intermediate	64 (45.1)
Poor	29 (20.4)
**PIV, high**	71 (50.0)
**PILE, high**	51 (35.9)
**SII, high**	71 (50.0)
**PLR, high**	71 (50.0)

Continuous variables are presented as mean ± SD (leukocytes, hemoglobin) or median (IQR); categorical variables as *n* (%). CRP was missing in 8 patients (5.6%); the median is based on *n* = 134. TPS, tumor proportion score; dNLR, derived neutrophil-to-lymphocyte ratio; ECOG, Eastern Cooperative Oncology Group; IQR, interquartile range; LIPI, Lung Immune Prognostic Index; NLR, neutrophil-to-lymphocyte ratio; PD-L1, programmed death-ligand 1; PILE, PIV—LDH–-ECOG-based score; PIV, pan-immune–inflammation value; PLR, platelet-to-lymphocyte ratio; SD, standard deviation; SII, systemic immune–inflammation index.

**Table 2:** Distribution of baseline characteristics by LIPI group.

Variable	Good (*n* = 49)	Intermediate (*n* = 64)	Poor (*n* = 29)	*p*	Cramér’s V (95% CI)
**Age ≥ 65, present**	32 (65.3)	31 (48.4)	11 (37.9)	**0.047**	0.207 (0.082–0.371)
**Sex**				0.405	0.113 (0.028–0.314)
Female	5 (10.2)	8 (12.5)	6 (20.7)		
Male	44 (89.8)	56 (87.5)	23 (79.3)		
**ECOG performance status ^‡^**				0.129	0.158 (0.090–0.302)
0	35 (71.4)	43 (67.2)	13 (44.8)		
1	12 (24.5)	16 (25.0)	14 (48.3)		
2	2 (4.1)	5 (7.8)	2 (6.9)		
**Smoking status ^‡^**				0.750	0.084 (0.056–0.223)
Current smoker	25 (51.0)	38 (59.4)	17 (58.6)		
Former smoker	19 (38.8)	20 (31.2)	11 (37.9)		
Never smoker	5 (10.2)	6 (9.4)	1 (3.4)		
**Histology ^‡^**				0.219	0.143 (0.081–0.279)
Adenocarcinoma	19 (38.8)	25 (39.1)	18 (62.1)		
Squamous cell carcinoma	28 (57.1)	34 (53.1)	10 (34.5)		
Other	2 (4.1)	5 (7.8)	1 (3.4)		
**Metastatic disease type**				0.189	0.153 (0.045–0.323)
De novo metastatic	29 (59.2)	43 (67.2)	23 (79.3)		
Recurrent metastatic	20 (40.8)	21 (32.8)	6 (20.7)		
**Liver metastasis, present**	5 (10.2)	17 (26.6)	7 (24.1)	0.087	0.185 (0.069–0.334)
**Bone metastasis, present**	13 (26.5)	25 (39.1)	19 (65.5)	**0.003**	0.286 (0.143–0.450)
**Brain metastasis, present**	6 (12.2)	14 (21.9)	4 (13.8)	0.353	0.121 (0.030–0.293)
**Adrenal metastasis, present**	10 (20.4)	18 (28.1)	9 (31.0)	0.515	0.097 (0.025–0.275)
**Nivolumab line, third**	4 (8.2)	7 (10.9)	5 (17.2)	0.469	0.103 (0.030–0.294)
**PD-L1 (≥1), present ^‡^**	19 (54.3)	23 (47.9)	9 (39.1)	0.528	0.110 (0.033–0.320)

Data are *n* (%); percentages are based on available data within each group. ^‡^ Fisher–Freeman–Halton test; other variables, Pearson chi-square test. Effect size is Cramér’s V (95% CI from 2000 bootstrap replicates). For the PD-L1 row, the denominator indicates patients with known PD-L1 within each group. Bold *p*-values indicate statistical significance (*p* ≤ 0.05). CI, confidence interval; LIPI, Lung Immune Prognostic Index; PD-L1, programmed death-ligand 1.

**Table 3:** Distribution of treatment response by LIPI group.

Response	Total (*n* = 142)	Good (*n* = 49)	Intermediate (*n* = 64)	Poor (*n* = 29)	*p*
**Complete response (CR)**	0 (0.0)	0 (0.0)	0 (0.0)	0 (0.0)	**0.003**
**Partial response (PR)**	42 (29.6)	21 (42.9)	17 (26.6)	4 (13.8)	
**Stable disease (SD)**	37 (26.1)	16 (32.7)	13 (20.3)	8 (27.6)	
**Progressive disease (PD)**	46 (32.4)	10 (20.4)	27 (42.2)	9 (31.0)	
**Not evaluable ^†^**	17 (12.0)	2 (4.1)	7 (10.9)	8 (27.6)	
**Objective response rate (ORR)**	42 (29.6) [22.7–37.5]	21 (42.9)	17 (26.6)	4 (13.8)	**0.019**
**Disease control rate (DCR)**	79 (55.6) [47.4–63.5]	37 (75.5)	30 (46.9)	12 (41.4)	**0.002**

Data are *n* (%); ORR and DCR are also given with Wilson 95% CI. *p*-values are from chi-square/Fisher–Freeman–Halton tests across LIPI groups. Response distribution Cramér’s V = 0.264 (0.189–0.403); ORR V = 0.236, DCR V = 0.293. ^†^ Patients without a formal RECIST 1.1 response assessment; the denominator for ORR/DCR is the whole cohort. No complete response was observed. Bold *p*-values indicate statistical significance (*p* ≤ 0.05). CI, confidence interval; DCR, disease control rate; LIPI, Lung Immune Prognostic Index; ORR, objective response rate.

**Table 4:** Overall survival nomogram: model coefficients and internal validation.

Component	Estimate (95% CI)
**Final model coefficients**	β/SE/HR (95% CI)
ECOG 1 (reference: 0)	−0.131/0.227/0.88 (0.56–1.37)
ECOG 2 (reference: 0)	**1.498/0.379/4.47 (2.13–9.40)**
Albumin (per 1 g/dL)	**−1.279/0.251/0.28 (0.17–0.46)**
LIPI intermediate (reference: good)	0.422/0.224/1.52 (0.98–2.36)
LIPI poor (reference: good)	**1.232/0.295/3.43 (1.92–6.12)**
**Discrimination and calibration**	Apparent/Optimism-corrected
C-statistic (Harrell)	0.752/0.742 (0.703–0.781)
Calibration slope	0.909 (0.681–1.136)
Brier score (12 months, IPCW)	0.153
**Observed overall survival across risk groups**	
6 months	25–92%
12 months	10–82%
18 months	6–62%

The nomogram is based on a Cox model for overall survival including ECOG performance status (categorical: 0/1/2), albumin, and LIPI. β, coefficient; SE, standard error; CI, confidence interval; ECOG, Eastern Cooperative Oncology Group; HR, hazard ratio; IPCW, inverse probability of censoring weighting; LIPI, Lung Immune Prognostic Index. Internal validation used 1000 bootstrap replicates with optimism correction. The calibration slope is optimism-corrected (ideal = 1.00). The Brier score was computed at 12 months with censoring weighting (lower = better). Risk groups are tertiles of the linear predictor; the best-to-worst group Kaplan–Meier range is given at each time point. HRs whose 95% CI excludes 1.00 are shown in bold.

## Data Availability

The data supporting the findings of this study are available from the corresponding author upon reasonable request. The data are not publicly available because of privacy and ethical restrictions.

## Supplementary Materials

The following supporting information can be downloaded at: https://www.mdpi.com/article/10.3390/jcm15176869/s1; Table S1: STROBE Statement—checklist for cohort studies; Table S2: TRIPOD Checklist—prediction model development with internal validation; Table S3: Systemic treatment history before and after nivolumab.; Table S4: Reasons for nivolumab discontinuation; Table S5: Baseline characteristics of patients without formal RECIST 1.1 response assessment (not evaluable) compared with response-evaluable patients; Table S6: Median survival and landmark survival rates by LIPI group; Figure S1: Kaplan–Meier estimates of overall survival according to PIV (A), PILE (B), SII (C) and PLR (D); Figure S2: Calibration of the nomogram for 6-, 12-, and 18-month overall survival; Table S7: Cox regression analyses for overall and progression-free survival; Table S8: Univariable association of PD-L1 status (including unknown) with survival; Table S9: Overall survival jointly stratified by LIPI risk group and PD-L1 status.

## Abbreviations

The following abbreviations are used in this manuscript:

LIPI	Lung Immune Prognostic Index
NSCLC	Non-small cell lung cancer
ICIs	Immune checkpoint inhibitors
STROBE	Strengthening the Reporting of Observational Studies in Epidemiology
TRIPOD	Transparent Reporting of a multivariable prediction model for Individual Prognosis or Diagnosis
LDH	Lactate dehydrogenase
dNLR	Neutrophil–lymphocyte ratio
ECOG	Eastern Cooperative Oncology Group
PIV	Pan-immune–inflammation value
PILE	PIV-–LDH-–ECOG-based score
SII	Systemic immune–inflammation index
PLR	Platelet-to-lymphocyte ratio
OS	Overall survival
PFS	Progression-free survival
ORR	Objective response rate
DCR	Disease control rate
PD-L1	Programmed Death-Ligand 1
CI	Confidence Interval
HR	Hazard Ratio
IQR	Interquartile Range
RECIST	Response Evaluation Criteria in Solid Tumors
