# Supplementary material for: Prognostic Value of the Lung Immune Prognostic Index and an ECOG–Albumin–LIPI Nomogram in Metastatic NSCLC Patients Treated with Second- or Third-Line Nivolumab
Source: J Clin Med. 2026 Sep 4;15(17):6869. doi: 10.3390/jcm15176869 (PMC13566715; doi:10.3390/jcm15176869)
Supplement: Supplementary file 1 [file jcm-15-06869-s001.zip › Supplementary Tables.pdf]

Table S1. STROBE Statement — checklist for cohort studies

Item	Recommendation	No.	Location in manuscript
**Title and abstract**	Indicate the study's design with a commonly used term in the title or the abstract; provide an informative and balanced summary	1	Title; Abstract
**Background/rationale**	Explain the scientific background and rationale for the investigation	2	Introduction, Section 1
**Objectives**	State specific objectives, including any prespecified hypotheses	3	Introduction, Section 1 (final paragraph)
**Study design**	Present key elements of study design early in the paper	4	Methods, Study Design and Ethical Approval
**Setting**	Describe the setting, locations, and relevant dates, including periods of recruitment and follow-up	5	Methods, Study Population; Figure 1
**Participants**	Give the eligibility criteria and the sources and methods of selection of participants; describe methods of follow-up	6	Methods, Study Population; Figure 1
**Variables**	Clearly define all outcomes, exposures, predictors, potential confounders, and effect modifiers; give diagnostic criteria, if applicable	7	Methods, Variables and Endpoints
**Data sources/measurement**	For each variable of interest, give sources of data and details of methods of assessment	8	Methods, Data Collection
**Bias**	Describe any efforts to address potential sources of bias	9	Methods, Statistical Analysis (multiple imputation; sensitivity analyses); Discussion (limitations)
**Study size**	Explain how the study size was arrived at	10	Methods, Study Population; Figure 1 (all eligible patients in the study period)
**Quantitative variables**	Explain how quantitative variables were handled in the analyses; describe which groupings were chosen and why	11	Methods, Variables and Endpoints; Statistical Analysis
**Statistical methods**	Describe all statistical methods, including those used to control for confounding; methods for subgroups, missing data, and sensitivity analyses	12	Methods, Statistical Analysis
**Participants (results)**	Report numbers of individuals at each stage of the study; give reasons for non-participation; consider use of a flow diagram	13	Results, Section 3.1; Figure 1
**Descriptive data**	Give characteristics of study participants and information on exposures and potential confounders; indicate number of participants with missing data	14	Results, Section 3.1; Table 1
**Outcome data**	Report numbers of outcome events or summary measures over time	15	Results, Sections 3.3 and 3.4; Tables 3 and S6
**Main results**	Give unadjusted estimates and, if applicable, confounder-adjusted estimates and their precision; report category boundaries for continuous variables	16	Results, Section 3.4; Table S6; Figure 4
**Other analyses**	Report other analyses done — subgroups, interactions, and sensitivity analyses	17	Results, Section 3.4; Tables S4 and S5; Figure S1
**Key results**	Summarise key results with reference to study objectives	18	Discussion, Section 4 (opening paragraphs)
**Limitations**	Discuss limitations of the study, taking into account sources of potential bias or imprecision	19	Discussion, Section 4 (limitations paragraph)
**Interpretation**	Give a cautious overall interpretation of results considering objectives, limitations, multiplicity of analyses, and other relevant evidence	20	Discussion, Section 4
**Generalisability**	Discuss the generalisability (external validity) of the study results	21	Discussion, Section 4 (limitations paragraph)
**Funding**	Give the source of funding and the role of the funders for the present study	22	Funding statement (back matter)

STROBE, Strengthening the Reporting of Observational Studies in Epidemiology. Item numbers follow the STROBE checklist for cohort studies; sub-items applicable only to case-control or cross-sectional designs are omitted.

Table S2. TRIPOD Checklist — prediction model development with internal validation

Item	Recommendation	No.	Location in manuscript
**Title**	Identify the study as developing and/or validating a multivariable prediction model, the target population, and the outcome	1	Title
**Abstract**	Provide a summary of objectives, study design, setting, participants, sample size, predictors, outcome, statistical analysis, results, and conclusions	2	Abstract
**Background**	Explain the medical context and rationale for developing the multivariable prediction model	3a	Introduction, Section 1
**Objectives**	Specify the objectives, including whether the study describes the development or validation of the model	3b	Introduction, Section 1 (final paragraph)
**Source of data**	Describe the study design or source of data, separately for the development and validation data sets	4a	Methods, Study Design and Ethical Approval
**Participants (dates)**	Specify the key study dates, including start of accrual, end of accrual, and end of follow-up	4b	Methods, Study Population; Results 3.3 (follow-up)
**Participants (setting)**	Specify key elements of the study setting, including number and location of centres	5a	Methods, Study Population
**Eligibility**	Describe eligibility criteria for participants	5b	Methods, Study Population
**Treatment**	Give details of treatments received, if relevant	5c	Methods, Study Population (second- or later-line nivolumab)
**Outcome**	Clearly define the outcome that is predicted by the prediction model, including how and when assessed	6a	Methods, Variables and Endpoints (OS definition)
**Outcome blinding**	Report any actions to blind assessment of the outcome to be predicted	6b	Not applicable — OS is an objective endpoint ascertained from records
**Predictors**	Clearly define all predictors used in developing the multivariable prediction model, including how and when they were measured	7a	Methods, Variables and Endpoints (ECOG PS, albumin, LIPI at nivolumab initiation)
**Predictor blinding**	Report any actions to blind assessment of predictors for the outcome and other predictors	7b	Not applicable — retrospective record-based assessment
**Sample size**	Explain how the study size was arrived at	8	Methods, Study Population (all eligible patients); events-per-variable reported in Statistical Analysis
**Missing data**	Describe how missing data were handled, with details of any imputation method	9	Methods, Statistical Analysis (multiple imputation for PD-L1; complete-case sensitivity analysis)
**Statistical analysis**	Describe how predictors were handled, model-building procedures, and internal validation	10a–10b	Methods, Statistical Analysis (prespecified covariates; bootstrap internal validation)
**Model performance**	Specify all measures used to assess model performance	10d	Methods, Statistical Analysis (C-index, calibration slope and intercept, Brier score)
**Participants (results)**	Describe the flow of participants through the study; consider a diagram	13a	Figure 1; Results, Section 3.1
**Model development**	Report the number of participants and outcome events in the analysis; report the unadjusted and adjusted associations	13b–14	Results, Section 3.4; Table S6
**Model specification**	Present the full prediction model to allow predictions for individuals; explain how to use it	15a–15b	Figure 5 (nomogram); Table 4 (model coefficients and performance)
**Model performance (results)**	Report performance measures with confidence intervals for the prediction model	16	Results, Section 3.4; Table 4; Figure S2 (calibration)
**Limitations**	Discuss any limitations of the study, such as a nonrepresentative sample or overfitting	18	Discussion, Section 4 (limitations paragraph; external validation not performed)
**Interpretation and implications**	Give an overall interpretation and discuss the potential clinical use of the model and implications for future research	19b–20	Discussion, Section 4; Conclusions, Section 5
**Supplementary information**	Provide information about the availability of supplementary resources	21	Supplementary Materials paragraph
**Funding**	Give the source of funding and the role of the funders	22	Funding statement (back matter)

TRIPOD, Transparent Reporting of a multivariable prediction model for Individual Prognosis Or Diagnosis. Items specific to external validation studies (10c, 10e, 12, 13c, 17, 19a) are omitted as no external validation cohort was used; this is stated as a limitation in the Discussion. Item 11 (risk groups) is not applicable, as no risk-group stratification was performed.

Table S3. Systemic treatment history before and after nivolumab.

Treatment history	n (%)
**First-line regimen, n (%)**	
Carboplatin–paclitaxel	83 (58.5)
Cisplatin–gemcitabine	36 (25.4)
Carboplatin–gemcitabine	11 (7.7)
Carboplatin–pemetrexed	7 (4.9)
Cisplatin–vinorelbine	5 (3.5)
**Prior exposure, n (%)**	
Chemo-immunotherapy	0 (0.0)
Any immune checkpoint inhibitor	0 (0.0)
**Second-line regimen among third-line nivolumab (n = 16), n (%)**	
Pemetrexed	6 (37.5)
Carboplatin–paclitaxel	5 (31.3)
Docetaxel	5 (31.3)
**Post-nivolumab regimen sequence, n (%)**	
No systemic treatment	82 (57.7)
Docetaxel	29 (20.4)
Docetaxel, then vinorelbine	6 (4.2)
Docetaxel, then gemcitabine, then vinorelbine	6 (4.2)
Gemcitabine	6 (4.2)
Docetaxel, then pemetrexed	4 (2.8)
Docetaxel, then gemcitabine	4 (2.8)
Pemetrexed	3 (2.1)
Docetaxel, then gemcitabine, then pemetrexed	2 (1.4)
**Number of post-nivolumab systemic lines, n (%)**	
0	82 (57.7)
1	38 (26.8)
2	14 (9.9)
3	8 (5.6)

All switches to nivolumab followed documented progression on the preceding treatment line. The median interval from initiation of the most recent prior treatment line to nivolumab was 7.0 months (IQR 4.8–11.9) overall: 6.9 months (IQR 4.7–11.0) for second-line and 7.8 months (IQR 6.1–20.4) for third-line nivolumab. IQR, interquartile range. Percentages are based on all 142 patients unless otherwise indicated.

Table S4. Reasons for nivolumab discontinuation (n = 142).

Reason for discontinuation	Patients, n (%)
**Disease progression**	80 (56.3)
**Death unrelated to disease progression**	41 (28.9)
**Ongoing treatment at data lock**	15 (10.6)
**Immune-related pneumonitis**	3 (2.1)
**Patient decision**	2 (1.4)
**Uncontrolled hemoptysis**	1 (0.7)

Data are n (%). Reasons were recorded by the treating physician at the time of discontinuation; patients with ongoing treatment had not discontinued nivolumab at the data-lock date.

Table S5. Baseline characteristics of patients without formal RECIST 1.1 response assessment (not evaluable) compared with response-evaluable patients.

Characteristic	Not evaluable (n = 17)	Evaluable (n = 125)	p
**Age (years), median (IQR)**	65.2 (60.1–68.4)	65.7 (60.2–69.7)	0.758
**Sex, n (%)**			
Male	14 (82%)	109 (87%)	
Female	3 (18%)	16 (13%)	0.702
**ECOG, n (%)**			
0	5 (29%)	86 (69%)	
1	9 (53%)	33 (26%)	
2	3 (18%)	6 (5%)	**0.004**
**LIPI risk group, n (%)**			
Good	2 (12%)	47 (38%)	
Intermediate	7 (41%)	57 (46%)	
Poor	8 (47%)	21 (17%)	**0.008**
**Albumin (g/dL), median (IQR)**	3.30 (3.10–3.80)	4.00 (3.70–4.30)	**<0.001**
**Liver metastasis, n (%)**	5 (29%)	24 (19%)	0.342

ECOG, Eastern Cooperative Oncology Group; IQR, interquartile range; LIPI, Lung Immune Prognostic Index; OS, overall survival; RECIST, Response Evaluation Criteria in Solid Tumours. Continuous variables were compared with the Mann–Whitney U test; sex and liver metastasis with Fisher's exact test; ECOG and LIPI with the chi-square test. Reasons for non-evaluability: early death before the first response assessment (n = 14), transfer to another center (n = 2), and loss to follow-up (n = 1). All 17 patients died during follow-up (median overall survival 0.9 months). Significant p values are shown in bold.

Table S6. Median survival and landmark survival rates by LIPI group

Outcome	Good (n = 49)	Intermediate (n = 64)	Poor (n = 29)	Log-rank p
**Overall survival**				**<0.001**
Median, months (95% CI)	19.3 (12.7–26.7)	8.0 (4.6–11.8)	3.1 (1.5–5.1)	
6-month rate, % (95% CI)	78 (63–87)	58 (45–69)	24 (11–41)	
12-month rate, % (95% CI)	67 (52–79)	36 (24–48)	10 (3–24)	
24-month rate, % (95% CI)	43 (29–56)	13 (6–22)	0 (0–0)	
**Progression-free survival**				**<0.001**
Median, months (95% CI)	8.6 (4.4–16.6)	3.1 (2.8–4.1)	2.5 (1.4–3.4)	
6-month rate, % (95% CI)	57 (42–70)	23 (14–34)	17 (6–33)	
12-month rate, % (95% CI)	43 (29–56)	13 (6–22)	3 (0–15)	
24-month rate, % (95% CI)	26 (15–39)	6 (2–14)	0 (0–0)	

Kaplan-Meier method; groups were compared with the log-rank test. Median and landmark (6, 12, 24 months) rates are given with 95% CI. NR, not reached (median not reached or confidence limit undefined). CI, confidence interval; LIPI, Lung Immune Prognostic Index. **Bold p values indicate statistical significance ($p \leq 0.05$).**

Table S7. Cox regression analyses for overall and progression-free survival

Variables	PFS univariable HR (95% CI)	p	PFS multivariable HR (95% CI)	p	OS univariable HR (95% CI)	p	OS multivariable HR (95% CI)	p
**Age (per 10-year increase)**	0.99 (0.81–1.22)	0.941	1.02 (0.79–1.32)	0.857	0.97 (0.80–1.18)	0.760	0.91 (0.71–1.19)	0.500
**Sex** *(male vs female)*	0.69 (0.42–1.13)	0.139	—	—	0.74 (0.45–1.20)	0.221	—	—
**ECOG** *(1 vs 0)*	1.20 (0.82–1.76)	0.351	0.90 (0.57–1.42)	0.646	1.37 (0.94–2.01)	0.105	0.88 (0.55–1.41)	0.599
**ECOG** *(2 vs 0)*	2.90 (1.46–5.79)	**0.002**	2.45 (1.15–5.20)	**0.020**	5.03 (2.45–10.35)	**<0.001**	4.94 (2.27–10.75)	**<0.001**
**Smoking** *(former vs current)*	0.81 (0.56–1.16)	0.250	—	—	0.80 (0.55–1.15)	0.225	—	—
**Smoking** *(never vs current)*	0.76 (0.40–1.46)	0.414	—	—	0.61 (0.32–1.17)	0.134	—	—
**Histology** *(squamous vs adeno)*	0.92 (0.65–1.30)	0.639	0.99 (0.67–1.46)	0.955	1.01 (0.71–1.43)	0.957	1.27 (0.85–1.90)	0.245
**Histology** *(other vs adeno)*	0.83 (0.36–1.88)	0.648	1.47 (0.60–3.59)	0.395	0.69 (0.30–1.57)	0.378	1.30 (0.50–3.40)	0.596
**Metastatic type** *(recurrent vs de novo)*	0.76 (0.53–1.10)	0.150	—	—	0.86 (0.59–1.24)	0.423	—	—
**Liver metastasis** *(present vs absent)*	1.67 (1.09–2.56)	**0.019**	1.39 (0.88–2.19)	0.157	1.45 (0.95–2.21)	0.088	0.95 (0.59–1.51)	0.814
**Bone metastasis** *(present vs absent)*	1.48 (1.04–2.12)	**0.030**	—	—	1.24 (0.87–1.77)	0.234	—	—
**Brain metastasis** *(present vs absent)*	1.20 (0.76–1.90)	0.436	—	—	1.10 (0.69–1.75)	0.683	—	—
**Adrenal metastasis** *(present vs absent)*	1.07 (0.71–1.60)	0.758	—	—	1.16 (0.77–1.75)	0.471	—	—
**PD-L1** *(≥1 vs <1)*‡	0.60 (0.40–0.91)	**0.017**	0.72 (0.48–1.07)	0.100	0.64 (0.42–0.96)	**0.033**	0.77 (0.51–1.17)	0.227
**Nivolumab line** *(third vs second)*	1.22 (0.72–2.07)	0.461	—	—	1.22 (0.72–2.07)	0.454	—	—
**Albumin (per 1 g/dL increase)**	0.34 (0.23–0.51)	**<0.001**	0.47 (0.29–0.75)	**0.002**	0.21 (0.13–0.31)	**<0.001**	0.27 (0.16–0.45)	**<0.001**
**LIPI** *(intermediate vs good)*	1.50 (1.05–2.14)	**0.025**	1.85 (1.17–2.93)	**0.008**	1.34 (0.94–1.91)	0.107	1.51 (0.93–2.43)	0.094
**LIPI** *(poor vs good)*	2.32 (1.52–3.53)	**<0.001**	2.82 (1.54–5.14)	**<0.001**	3.31 (2.14–5.11)	**<0.001**	3.74 (1.95–7.15)	**<0.001**
**PIV** *(high vs low)*§	1.43 (1.01–2.03)	**0.047**	—	—	1.84 (1.29–2.61)	**<0.001**	—	—
**PILE** *(high vs low)*§	2.34 (1.62–3.38)	**<0.001**	—	—	2.81 (1.94–4.06)	**<0.001**	—	—
**SII** *(high vs low)*§	1.25 (0.88–1.77)	0.220	—	—	1.70 (1.19–2.42)	**0.003**	—	—
**PLR** *(high vs low)*§	1.32 (0.93–1.87)	0.125	—	—	1.41 (0.99–2.01)	0.055	—	—

HR, hazard ratio; CI, confidence interval. The multivariable Cox model included age, ECOG, histology, liver metastasis, PD-L1, albumin and LIPI (10 degrees of freedom; events-per-variable ≈12.6). In the multivariable model PD-L1 was imputed by multiple imputation (predictive mean matching, m=30) and coefficients were pooled by Rubin's rules; complete-case sensitivity analysis (n=106) was consistent. The proportional hazards assumption was verified using scaled Schoenfeld residuals (no violation); multicollinearity was excluded with VIF < 2. ‡The univariable PD-L1 analysis was based on 106 patients with known PD-L1. §PIV, PILE, SII and PLR were assessed only at the univariable level as exploratory analyses. In the multivariable OS model, ECOG (2 vs 0), albumin and LIPI (poor vs good) remained significant after Benjamini–Hochberg correction. Bold p values indicate statistical significance ($p \leq 0.05$). LIPI, Lung Immune Prognostic Index; LDH, lactate dehydrogenase; OS, overall survival; PFS, progression-free survival; VIF, variance inflation factor; ECOG, Eastern Cooperative Oncology Group; PD-L1, programmed death-ligand 1; PIV, pan-immune-inflammation value; PILE, PIV-LDH-ECOG based score; SII, systemic immune-inflammation index; PLR, platelet-to-lymphocyte ratio.

Table S8. Univariable association of PD-L1 status (including unknown) with survival

PD-L1 category	OS HR (95% CI)	p	PFS HR (95% CI)	p
**< 1 (reference)**	1.00 (ref)	—	1.00 (ref)	—
**≥ 1**	0.62 (0.41–0.94)	**0.025**	0.58 (0.38–0.88)	**0.010**
**Unknown**	0.77 (0.50–1.19)	0.239	0.69 (0.44–1.07)	0.095

To assess potential bias from missing PD-L1 data, the unknown category was modelled separately (reference <1; whole cohort, n=142). The lack of a significant difference between the unknown and reference categories supports that missingness is not strongly associated with survival. CI, confidence interval; HR, hazard ratio; OS, overall survival; PFS, progression-free survival. **Bold p values indicate statistical significance ($p \leq 0.05$).**

Table S9. Overall survival jointly stratified by LIPI risk group and PD-L1 status (patients with known PD-L1, n = 106).

Stratum	n	Median OS (months)	HR (95% CI)
**PD-L1 <1% (n=55)**			
Good	16	9.1	Reference
Intermediate	25	10.1	1.34 (0.68–2.64)
Poor	14	3.1	**3.45 (1.59–7.49)**
Log-rank p		**0.002**	
**PD-L1 ≥1% (n=51)**			
Good	19	24.9	Reference
Intermediate	23	6.0	**2.28 (1.10–4.70)**
Poor	9	3.6	**5.29 (2.13–13.11)**
Log-rank p		**0.001**	

CI, confidence interval; HR, hazard ratio; LIPI, Lung Immune Prognostic Index; OS, overall survival; PD-L1, programmed death-ligand 1. HRs are from stratum-specific Cox models with the good LIPI group as reference; p-values are from the log-rank test across LIPI groups within each PD-L1 stratum. The multiplicative LIPI × PD-L1 interaction, tested in the full multivariable model under multiple imputation (D1 pooled Wald test, 2 df), was not significant ($F = 0.48$, $p = 0.620$). HRs whose 95% CI excludes 1.00 and significant p values are shown in bold.
